# Electrospun PEO/PEDOT:PSS Nanofibers for Wearable Physiological Flex Sensors

**DOI:** 10.3390/s21124110

**Published:** 2021-06-15

**Authors:** Eve Verpoorten, Giulia Massaglia, Candido Fabrizio Pirri, Marzia Quaglio

**Affiliations:** 1 Department of Applied Science and Technology, DISAT, Politecnico di Torino, 10129 Torino, Italy; giulia.massaglia@polito.it (G.M.); fabrizio.pirri@polito.it (C.F.P.); 2 Center for Sustainable Future Technologies, Italian Institute of Technology, 10144 Turin, Italy

**Keywords:** blend polymeric solution, piezoresistivity, flex mechanical sensor, pH sensor, wearable physiological flex sensors

## Abstract

Flexible sensors are fundamental devices for human body monitoring. The mechanical strain and physiological parameters coupled sensing have attracted increasing interest in this field. However, integration of different sensors in one platform usually involves complex fabrication process-flows. Simplification, even if essential, remains a challenge. Here, we investigate a piezoresistive and electrochemical active electrospun nanofibers (NFs) mat as the sensitive element of the wearable physiological flex sensing platform. The use of one material sensitive to the two kinds of stimuli reduces the process-flow to two steps. We demonstrate that the final NFs pH-Flex Sensor can be used to monitor the deformation of a human body joint as well as the pH of the skin. A unique approach has been selected for pH sensing, based on Electrochemical Impedance Spectroscopy (EIS). A linear dependence of the both the double layer capacitance and charge transfer re-sistance with the pH value was obtained by EIS, as well as a linear trend of the electrical resistance with the bending deformation. Gauge factors values calculated after the bending test were 45.84 in traction and 208.55 in compression mode, reflecting the extraordinary piezoresistive behavior of our nanostructured NFs.

## 1. Introduction

Flexible and wearable devices able to sense bending are more and more developed for applications such as smart clothing, rehabilitation, prosthetic limbs, sport and research [1,2,3,4,5,6,7,8,9]. During the last years, flexible devices to sense both mechanical strain and physiological parameters are attracting interest to design novel, robust and low-cost systems for personalized medicine [10,11,12,13,14,15,16]. Since the contractile activity of the muscles builds-up acid in muscle cells during exercise, the monitoring of sweat pH plays a crucial role to define the health state of the body, leading thus to correlate it to the physical activity [17,18,19]. For example, in an out-of-the-lab perspective, personalized rehabilitation therapy can include a multisensing platform able to sense the angular deformation as well as the pH, which allows the direct monitoring of muscle fatigue during training of body joints. For this purpose, the combination of sweat-pH and joint bending information obtained by stretchable and pH-sensitive flex sensors are of huge interest for multisensing systems. Moreover, the combination of a flex and pH sensing platform is also useful in a context of health monitoring. Indeed, since the pH is also affected by the presence of diseases such as cystic fibrosis, dehydration, diabetes and cancer [17,18,19], the multisensing system, based on flex sensor and pH sensor, plays a crucial role to establish if the pH changes are due to the physical activity or not, thus preventing the alert for a disease. In particular, in this work, we studied the possibility to apply directly the sensor on the skin and near of the joint, enabling the pH measurement to give information about the health state of the patient during some physical movements. As the pH range goes from 4 to 6, we decided to test our device extending this range of pH of an amount of 2 above and below the limits (i.e., 2 to 8). The goal for the pH sensor was to be able to give the alarm for deeper investigation and this extension of the range is more than enough to perform this achievement. Usually, multisensing platforms are composed of a flexible substrate (e.g., polydimethylsiloxane—PDMS, textiles, Kapton^®^) and a sensitive element that can be made of nanomaterials, intrinsically conductive polymers (ICPs), conductive inks or optical fibers. [4,5,6,7,8,9] Two-sensing units on one single platform often requires long and complicated fabrication process-flows, unless if the same sensitive material is used for the two units [2,3]. In this work, we proposed a multisensing platform based on the common sensitive element able to monitor the two signals, which implies one single and simple process flow for the whole platform. In this particular goal, ICPs are selected as active materials. ICPs have a tremendous potential for application in flexible wearable sensing [20] and among them, poly(3,4-ethylenedioxythiophene) (PEDOT) doped with poly(styrene sulfonate) (PEDOT:PSS) is the most widely used due to its high stability, high electrical conductivity and excellent processability [21,22]. This polymer is usually studied in a film form [22,23,24,25,26,27,28], but further improvement of ICPs in term of mechanical performance, adhesion with the substrate, water resistivity and conductivity is possible by shaping them in nanostructures [29], such as electrospun nanofibers (NFs) [4,30]. The inherent high porosity and high surface area to volume ratio of NFs can be exploited at their best to enhance the ICPs behavior.

In this present work, we first of all confirm that the good piezoresistive behavior of PEDOT:PSS [27,31] is maintained when it is blended with polyethylene oxide (PEO), moreover confirming that shaping the polymeric blend in nanofiber form is an effective strategy to improve the sensing response of the mechanical unit. We also demonstrate that the resulting PEO/PEDOT:PSS NFs are electrochemically active and suitable to work as the sensitive element of the electrochemical pH sensing unit. The two units are integrated into a platform based on a flexible substrate to ensure both ease of placement in contact to the body and ease of detection of both mechanical flexion and pH changes. The presence of PEO/PEDOT:PSS NFs as the active materials for both the sensing units introduces a unique opportunity to simplify the process flow for the whole platform fabrication, reducing both complexity and process time. Nevertheless, this choice introduces a new challenge in terms of detection. Indeed, since PEO/PEDOT:PSS NFs are placed on the flexible platform, it is important to ensure that the mechanical stress does not interfere with the detection of the electrochemical signals during pH analysis. Therefore, with the main aim to decouple the signals processed by the two sensors, we introduce a novel approach based on the electrochemical characterization, electrochemical impedance spectroscopy (EIS), for monitoring pH changes. EIS enables to characterize the electrochemical interfaces rather than the material itself, which ensures a better separation of the response of the device to the two kinds of stimuli: in the case of the mechanical stimuli, the bulk of the material is analyzed while in the case of the pH it is its surface that is characterized. 

The final NFs pH-Flex Sensor was obtained using a PDMS slab as the platform substrate. PDMS offers several advantages in terms of stability and flexibility in contact with the human body. Moreover, it simplifies the integration of electrochemical electrodes. The electrospun PEO/PEDOT:PSS NFs are stabilized by thermal treatment [4,32]. The electrochemical pH unit is based on commercially available electrodes that are integrated in the PDMS slab. The pH unit is conceived to be placed in direct contact with the skin, while the piezoresistive mechanical unit is placed on the opposite side and in a position that maximizes flection of the sensitive material. The sensitive electrospun NFs mats are directly collected on both surfaces of the PDMS substrate and laser ablation is used to pattern the NFs on the electrodes for pH sensing. Laser ablation allows removing NFs from the surface of both the counter and the reference electrodes, leaving nanostructured PEO/PEDOT:PSS on top of the working electrode only. 

The mechanical response is characterized through IV measurements while applying mechanical deformation. The extraordinary elastic behavior of the PDMS slab ensures optimal transfer of the mechanical deformation from the joint to the sensing NFs. Moreover, with the main purpose to evaluate the effectiveness of nanostructuration in the mechanical response, in this work we analyze two structures: (i) crosslinked NFs; (ii) mixed composition (MC), that is based on electrospun NFs immersed in a film-like arrangement. The MC structure shows an optimal mechanical behavior only in compression mode, while the crosslinked NFs demonstrate a better mechanical behavior in both compression and traction mode, as reflected in the values of the gauge factors (GFs) which are 45.84 and 208.55 for compression and traction, respectively. These results demonstrate the key role of the nanostructuration in sensing. Moreover, it is important to highlight how these latter values result to be higher than the one presented in the literature for non-nanostructurated PEDOT [24,28,31,33,34,35]. 

The pH sensing is based on the identification of the best fitting equivalent circuit to interpret the EIS spectra. Both the double layer capacitance and charge transfer resistance show an excellent linear dependence with the pH value, both in acid and basic environments, with an inversion corresponding to the same pH value of 5.8. Given a substantial lack of information in the literature about EIS-based measurements of pH in PEO/PEDOT:PSS sensing materials, a model is proposed to interpret the obtain results, evidencing the great potential of EIS for application in wearable sensors. 

## 2. Materials and Methods

### 2.1. NFs Synthesis and Design of the Platform

First, the PDMS (purchased from Silgard 184) was prepared as the flexible substrate for the sensors. This substrate was prepared using a 10:1 weight ratio of PDMS with respect to the curing agent. It was then crosslinked at 100 °C for 1 h on a hot-plate. A commercial carbon electrochemical 3-electrodes component (DropSens, Metrohm, Varese, Italy was integrated into this PDMS slab. The commercial component by DropSens is composed by 3 electrodes, the working and counter electrodes are made of gold, while the reference electrode is made of silver/silver chloride (Ag/AgCl). Then, the nanofibers were directly electrospun onto the substrate. The initial polymeric solution was made of 5 mL of PEDOT:PSS and a solution of PEO (5 wt%) in deionized water. It corresponds to a content of 4 wt% and 6.66 wt% of PEDOT and PSS, respectively, with respect to PEO. Polyethylene oxide (Mw = 600,000 Da) and PEDOT:PSS aqueous dispersion (1.3 wt%) were purchased from Signa Aldrich. The polymeric solution was stirred overnight at ambient temperature. Crosslinked NFs and MC were obtained by electrospinning using a NANON 01A apparatus (MECC Ltd.). A spinneret hosting a 27 gauge × 15 mm needle was powered by a high-power supply (HVU-30P100). The nanofiber mat was collected by applying a voltage of 15 kV at a working distance of 15 cm and for 15 min. A flow rate of 0.1 mL h^−1^ was chosen to obtain a good uniformity in the distribution of the NFs diameters. The MC was obtained imposing a working distance of 25 cm and using an isolating collector to obtain a floating potential. After the electrospinning process, the materials were treated to obtain crosslinked materials under inert atmosphere (360 sccm of N2 flow) in a Carbolite, VST furnace. The furnace was heated at a heating rate of 2.5 °C min^−1^. As reported in our previous work [32], the heating treatment is implemented with the definition of two steps: (i) the first one at 70 °C for 1 h to preserve the morphology of the starting nanostructures; (ii) the second step was conducted at 120 °C for 3 h to induce the crosslinking. After the electrospinning process and the thermal treatment, through laser ablation (Co2 Laser 10.6 μm, 30 W, Laser Marking System Slider), we are able to remove crosslinked NFs from the counter and reference electrodes of the commercial electrochemical 3-electrodes. In this way, we obtained binder free flex and pH sensors, where crosslinked NFs, representing the sensitive elements of the sensors, were directly integrated onto the flexible substrate of PDMS, which is able to confer stability and flexibility to the sensitive nanofiber mats. A paste made of PDMS, curing agent (10:1) and MWCNT (2 wt%) was spread in the flex sensing zone to create proper electrodes for electrical characterizations. 

### 2.2. Morphological, Physical and Chemical Characterizations

#### 2.2.1. Field Emission Scanning Electron Microscopy

A field emission scanning electron microscope (FESEM, Zeiss MERLIN), operating from 5 kV to 10 kV was used to evaluate the morphological properties of crosslinked NFs and the MC.

#### 2.2.2. Thickness Measurement 

The thickness of nanofiber mats was measured by a surface profilometer (TENCOR P-10).

#### 2.2.3. Electrical Characterizations

Electrical characterizations were conducted with the purpose to analyze the electrical behavior of the materials in bending mode thanks to a Keysight B2912A source measure unit. The measurements conducted were I-V. The voltage range applied was from −1 to 1 V, while the rate was 10 mV/s. The current flowing through the material thanks to its polarization was measured and the resistance interpolated. Three measurements were performed on each sample and the sample went back to rest position in between these measurements. The second Ohm’s law was applied to evaluate the resistivity of the materials. The studied parameters were directly plotted with Matlab and error bars based on the least square method were calculated. Results showed were obtained for at least 4 replica of each sample conditions. 

#### 2.2.4. Electrochemical Characterizations

EIS (frequency range: 0.1–100,000 Hz, perturbation amplitude: 0.01 V) measurements were performed with a Palmsens potentiostat, using a water-based solution at different pH. The pH of the solutions was controlled with a pH-meter (Hanna, HI5221) and was adjusted adding HCl or NaOH to deionized water. The EIS data was fitted into an equivalent circuit, chosen based on physical and chemical properties of the system, using an EIS Spectrum Analyser and the studied parameters were directly plotted with Matlab.

## 3. Results

**Figure 1:**
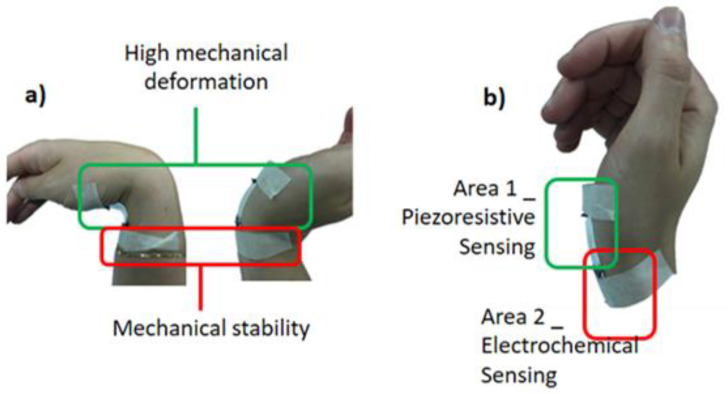
Application scheme of the NFs pH-Flex Sensors device, with highlight of (**a**) the two mechanical deformation zones and (**b**) the disposition of the sensors on the device based on these zones.

In this work, the application of PEO/PEDOT:PSS crosslinked NFs as the sensitive element for flex sensing coupled with pH sensing in human body joint monitoring was investigated. Since the two sensing units were integrated into a flexible platform, the identification of the zones of the joint in which each sensing unit should be placed was the very first challenge of the design of the system. Indeed, the piezoresistive and the pH sensors required different working conditions. The mechanical sensor needs to be exposed to the maximum deformation of the joint during flexion, while the pH unit has to be kept in contact to the skin, with the final goal to avoid any other perturbation. Figure 1a depicts the analysis of the mechanical deformation occurring in the wrist during flexion. To satisfy all requirements, two main regions have been identified as proposed in Figure 1b. Area 1 corresponds to the joint cavity, and it is suitable to offer the maximum deformation of the joint. Therefore, Area 1 is selected for piezoresistive sensing, guaranteeing a no-direct contact between the sensitive material and the skin. While Area 2, placed just beneath Area 1, offers a flat area of the skin not deformed by flexion. With this perspective, Area 2 is the choice for the disposition of the electrochemical sensor to monitor the acidity/basicity changes associated to sweat. To ensure optimal working condition for the pH sensor, it was disposed in direct contact with the skin in the zone offering the lowest mechanical deformation.

**Figure 2:**
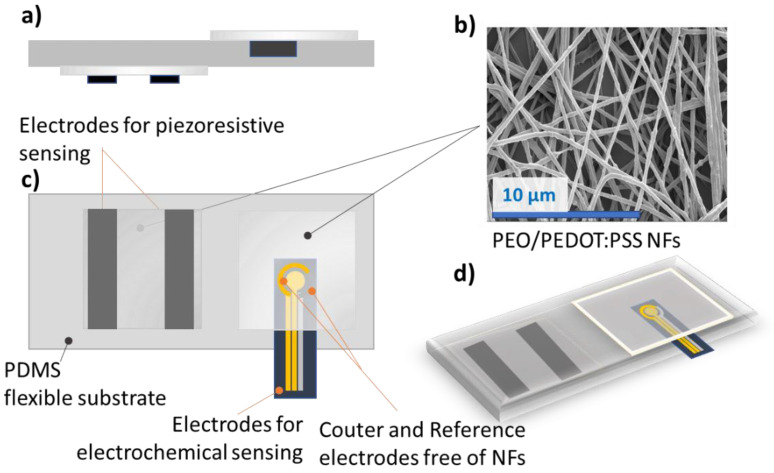
Design of the NFs pH-Flex Sensor. (**a**) cross view, (**b**) FESEM of the PEO/PEDOT:PSS crosslinked NFs, (**c**) top view of the device, where the position of counter and reference electrode is highlighted and (**d**) 3D view.

The great novelty of this device is based on the common sensitive element able to monitor the two signals, which implies one single and simple process flow for the whole platform. Figure 2 shows the design of the NFs pH-Flex Sensor and in particular, Figure 2a evidences the NFs disposition for direct contact with the skin (top) in opposition to the willing to isolate them from it (down). Through optimized thermal treatment, the crosslinking of PEO and PSS was performed, leading to an increase of the electrical conductivity, mechanical coupling with PDMS substrate and water resistance, as deeply demonstrated in our previous work [4,26,32]. Figure 2b shows the morphology of the NFs mat after thermal treatment, highlighting the preservation of the nanostructuration.

**Figure 3:**
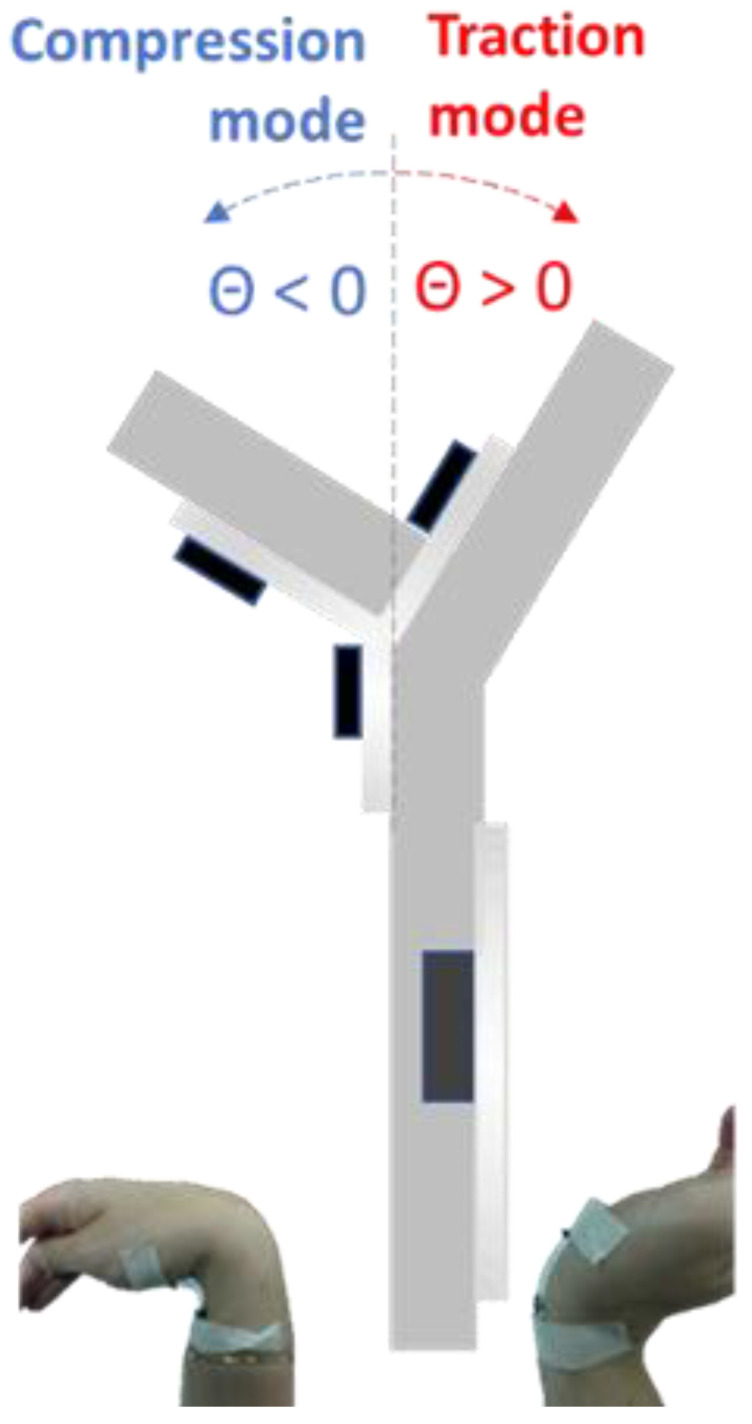
Model describing the bending and the determination of the compression/traction mode.

To understand the performances of the two sensors, current-voltage characterizations under bending of the sensor and EIS were performed for the flex and the pH sensor, respectively. The choice of the bending angle range was determined based on our observation of the maximal angles reachable for the human body joints, interesting to study in the context of health monitoring and rehabilitation. We used a model describing the bending and the determination of the compression/traction mode at which the sensor was exposed (Figure 3).

Table 1 summarizes these angles in function of the joint at which the sensor could be applied.

Therefore, within a range from −110° in compression and +110° in tension, we were able to monitor a huge range of articulation deformation. The direct application of the sensor on the skin and near of the joint enable the pH measurement to give information about the health state of the patient while it is moving the application. The pH range that we studied was from 2 to 8. 

**Figure 4:**
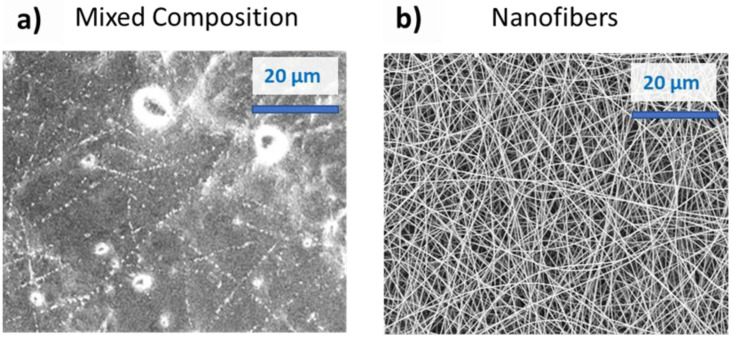
(**a**) FESEM of the PEO/PEDOT:PSS MC (electrospun nanofibers immersed in a film-like structure) and (**b**) FESEM of the PEO/PEDOT:PSS NFs.

The piezoresistive behavior of the flex sensor was investigated through current-voltage characterization submitting the samples to bending deformations. With the main aim to demonstrate the improvement obtained by implementation of electrospun NFs as sensitive material in flex-sensor, we compared two different structures: one called MC, made of electrospun NFs immersed in a film-like arrangement (Figure 4a) and the other one made of electrospun NFs, as confirmed by the morphological analysis sketched in Figure 4b. This latter MC structuration was obtained by increasing the working distance in the electrospinning process to 25 cm and using an isolating collector to obtain a floating potential.

**Figure 5:**
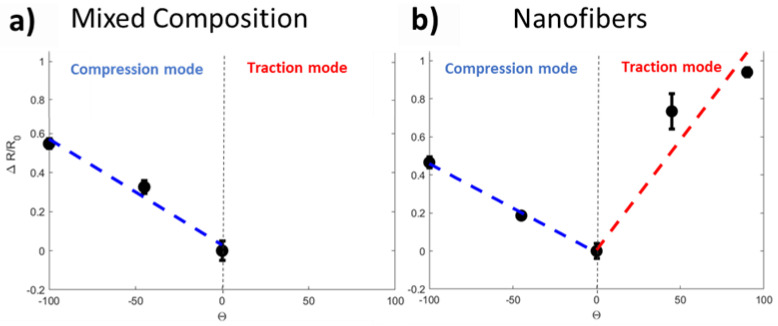
(**a**) resistance variation when bending the sample for the MC structuration and (**b**) resistance variation when bending the sample for the NFs.

Figure 5a,b report the variation in resistance (ΔR/R_0_) with the bending angle (determined as depicted in Figure 3) for both of two materials. R_0_ represent the situation in which the sensor is not deformed. 

**Figure 6:**
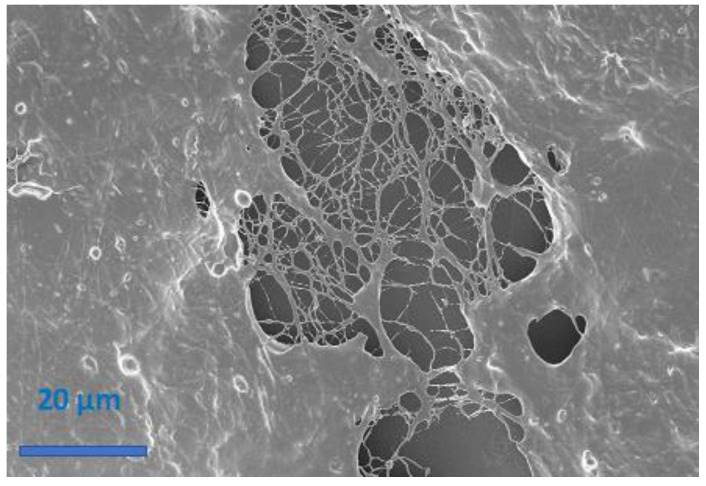
FESEM image of MC material submitted to bending in traction mode. The rupture of the material was evidenced in this image.

For the MC, a linear behavior was observed in compression mode. The slope of the curve, i.e., the sensitivity of the sensor, was about −0.0052 for this mode. In traction mode, we observed a sudden rupture of the MC that inhibited the response of the sensitive element, as confirmed by morphological analysis proposed in Figure 6. FESEM image highlights that bending deformation severely affects the film-like structure of the MC, while the NFs, immersed in this structure, are the last remaining components competing to fracture propagation. These latter results are in line with findings reported in the literature [33]. According to them, film form does not allow the measurement of angles in both compression and traction modes, because of the cracks induced in the sensitive material that limit the angle range of bending. To overcome these limits, NFs are proposed as sensitive materials. It is important to appreciate how for the NFs, a linear behavior was also observed in compression mode. The similar value of the curve slope (−0.0047 for the NFs and −0.0052 for the MC) suggest g no significative difference between the electrospun NFs and the MC for flex sensing in compression mode. However, in the case of the NFs a linear behavior was also achieved in traction mode, confirming the importance of the nanostructuration for flex sensing in a bimodal configuration. The NFs are mechanically more resistant to traction and therefore the material composed of exclusively nanofibers did not fracture in traction mode in the range of 0° to 100°.

All these obtained results allow confirming that the NFs form was the one that was suited best for the application in body joint monitoring. The gauge factors (GFs) obtained thanks to the model of Saggio et al. [34] amounted at 45.84 in traction mode and 208.55 in compression mode. The two very high values highlight how the nanostructuration of the material improves the sensitivity of the sensor in comparison with the GFs obtained for film-structure of PEDOT:PSS [24,28,31,35,36]. The sensor was also characterized for several bending cycles and the results are exposed in the Supporting Information. Thanks to the impressive sensitive behavior achieved by nanofibers structuration, an optimized multiplatform sensing based on nanomaterials are developed/designed to simultaneously measure the mechanical deformation and pH sensing. 

Since the NFs pH-Flex Sensor has the unique feature to use the same active material for both sensing pH changes and mechanical stimuli, in this work it was necessary to identify the electrochemical method able to ensure minimal disturbance due by the piezoresistive behavior of the material itself. For this reason, pH sensing was analyzed through EIS. Indeed, other electrochemical methods, such as potentiometric and amperometric methods, commonly used for electrochemical pH sensing, directly, the response of the material to the pH changes [37]. In our case, to decouple the signals processed by the two sensors, we choose EIS characterization, which is able to study the electrochemical interfaces between sensitive materials and solution rather than its bulk that is analyzed in the flex sensor. 

**Figure 7:**
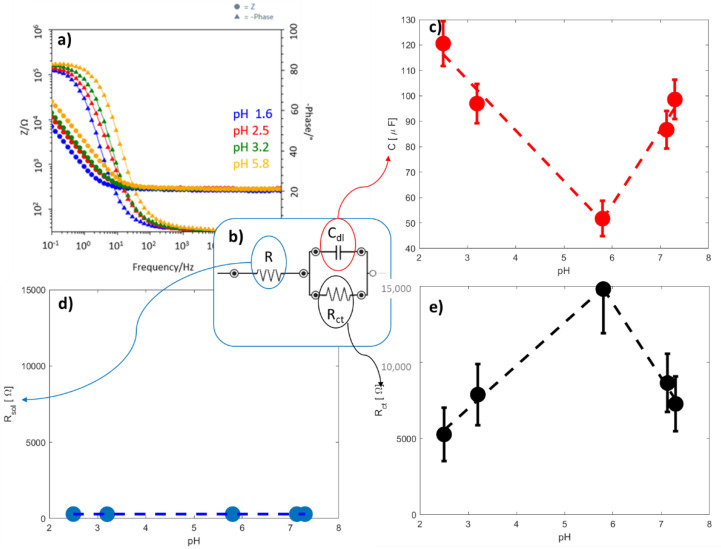
(**a**) EIS curves obtained with the sensor at 4 acidic pH values as example, (**b**) equivalent circuit used to determine electrochemical parameters, (**c**) double layer capacitance, (**d**) solution resistance and (**e**) charge transfer resistance variation of the electrochemical sensor with pH values.

A typical impedance response for the acid pH is showed on Figure 7a by Bode plots. A process is clearly visible at high frequency, which can be associated to the charge transfer at the electrode/solution interface. The impedance data were fitted through the equivalent circuit proposed in Figure 7b, composed by an ohmic resistance R in series with a Voigt element made of the parallel of a double-layer capacitance C_dl_ and a charge transfer resistance R_ct_. Table 2 reports the fitting values. The equivalent circuit was chosen based on the morphology of the interface of the sensor and the choice was confirmed by the literature [38,39].

The ohmic resistance R is due to two in-series resistances, i.e., R_sol_ and R_NFs_. The R_sol_ accounts for the resistance of the solution in direct contact with the interface of the sensor, and in the case of skin pH sensing it is the resistance of the sweat. R_NFs_ is the resistance of NFs used as sensing materials. The R_ct_ is the charge transfer resistance that describes the resistance of the electron transfer from the solution that is in contact with the electrode (i.e., the sweat) to the electrode itself. This resistance changes based on the nature of the interface double layer capacitance between the solution and the electrode. We, therefore, expected a change in the R_ct_ varying the pH. Finally, C_dl_ represents the capacitance that is formed at the surface of the electrode when the measurement is performed. This capacitance is also affected by the nature of the electrode and therefore we also expected a change in its value with the pH variation.

**Figure 8:**
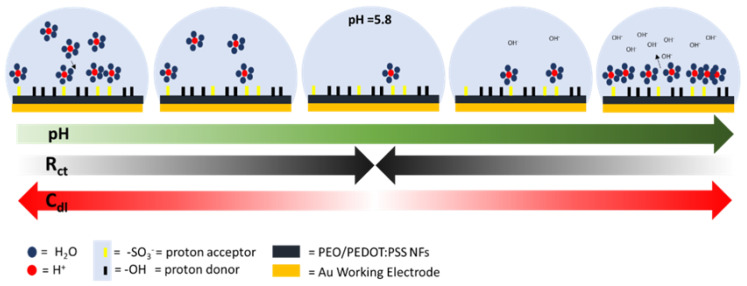
Proposed model of the interactions between the protons and the surface of the NFs at the interface between the material and the solution.

Figure 7c–e shows the results obtained for the pH sensing measurements. The R did not change when the solution pH was adjusted, as shown in Figure 7d. We did not expect that R_sol_ changed with pH as it is the same water-based solution for all the conditions and this absence of response in the global R showed us that the R_NFs_ was also stable with the pH. This result allows confirming that pH sensing of the nanofibers was concentrated on the surface of the material and not at its bulk. Two linear behaviors are observed for both the capacitance of the interface (C_dl,_ Figure 7c) and the charge transfer resistance (R_ct,_ Figure 7e). While the pH increases from 2 until 5.8, it was possible to observe how the C_dl_ decreases (*y* = −19.9*x* + 166) and R_ct_ increases (*y* = 2826*x* − 1517) in a linear way. After this point, with pH values in the range from 5.8 to 7, the tendency is reversed for both of the two parameters (C_dl_*: y* = 29.36*x* − 119; R_ct_: *y* = −4872*x* + 4309). It is interesting noticing that the peak value is the same for the two observed parameters, showing again that the observed effect is due to the material response to pH. As the healthy skin pH value is in the range of 4–6 [33], two zones can be created on the capacitance/R_ct_-pH curve: the one in which pH value corresponds to the physiological values, typical of healthy skin, (green zones) and the one in which further analysis needs to be performed because the skin is too acid/alkaline (red zones), as depicted in Figure 8. 

**Figure 9:**
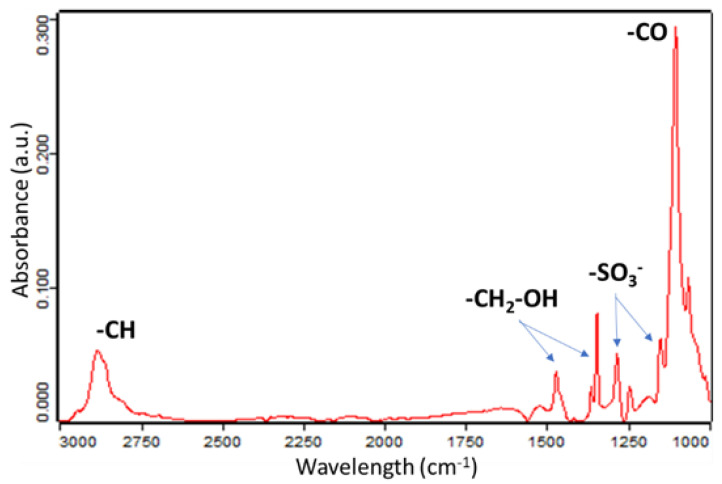
FTIR analysis of the PEO/PEDOT:PSS NFs.

Figure 8 provides a model to explain the electrochemical mechanisms behind this phenomenon. This model is based on the presence of -SO^3−^ and –OH groups on the surface of the NFs. These groups are presented on the surface of the NFs, as confirmed by performed FTIR analysis (Figure 9). As the group -SO^3-^ acts as a proton acceptor in acid environment, it attracts the protons in excess from the bulk of the solution towards the NFs, leading, thus, to increasing the C_dl_. While the pH increases, fewer protons are available and, consequently, the C_dl_ decreases until 5.8 when all the protons available are blocked near of the surface, removing them from the bulk of the solution. This configuration is the one at which the C_dl_ is the lowest. Indeed, while the pH increases towards basic environment, the –OH groups begin to release the protons to compensate the presence of the OH^-^ in the bulk of the solution and a capacitive layer is, therefore, formed. Higher the pH, higher the value of the C_dl_.

The R_ct_ depends directly on the nature of the interface between the solution and the NFs. The negatively charged surface of the NFs, due to the presence of the -SO^3−^ groups, acts as an intrinsic barrier for the electrons flow from the solution to the NFs, leading to a high R_ct_. With the presence of the protons near of the surface in basic/acid environment, the total negative charge of the surface is partially compensated, easing the electrons flow from one medium to the other, leading, thus, to a decrease in R_ct_ value.

## 4. Discussion

In this work, we demonstrated that, in addition to be a flex sensitive material, nanostructurated PEO/PEDOT:PSS is also an outstanding pH sensitive material. It is indeed an excellent choice for a multi-sensing platform aiming at analyzing body joint deformation combined with sweat pH, in the context of applications such as smart clothing, rehabilitation, prosthetic limbs and sport [1,2,3]. 

The point of strength of this designed wearable NFs pH-Flex Sensor, is the fact that the two sensing units are composed of the same material, nanostructurated PEO/PEDOT:PSS, integrated into the same multisensing platform. This approach enables the easier process flow for the fabrication of the device in comparison with the works presented in literature about the multisensing platform [40].

We demonstrated that EIS can be successfully selected as the electrochemical method for pH sensing in wearable applications. In this work we demonstrated that EIS characterization was an effective tool to decouple the mechanical and electrochemical signals processed by the two sensors. Exploiting the ability of EIS to characterize the electrochemical interfaces of a system, rather than the material itself, we succeeded in separating the response to pH, as analyzed by R_ct_ and C_dl_, to the resistance of the NFs.

We demonstrated the importance of the nanofiber form for the performances of the sensor. Indeed, we compared these performances with the one of two other material structures: the one that is the most present in literature, i.e., the film form [22,24,28,31,34], and a MC of film and electrospun NFs obtained through electrospinning with a floating potential. We demonstrated that both the mechanical resistance of the material and the performances of the sensor were better with the material in nanofiber form. Indeed, both in film form and in the MC, a rupture of the sensible material can be observed in traction mode. However, the electospun NFs did not break for the same angular deformations showing a higher mechanical resistance. About the performances of the sensor, the gauge factors obtained with the NFs were 45.84 in traction and 208.55 in compression mode. This result shows the important role of the nanostructuration since the gauge factors presented in literature for films are up to 17.8 [22,24,28,31,34]. We therefore emphasize the importance of the electrospinning technique in this work.

We also demonstrated the importance of the collocation and the design of this multisensing platform. Indeed, the signals obtained for each of the sensor should be free of disturbance coming from the other unit or a stimulus that is not the one that we want to analyze. With a proper collocation of the platform and a proper design, the presence of the two sensors on the same substrate allow easier remote measurements, easier manipulations and therefore increases the applications in a context out-of-the-lab for personalization of the health monitoring.

Indeed, the optimized wearable physiological flex sensor is able to directly and simultaneously provide flex sensing with sweat pH sensing, allowing the direct monitoring of muscle fatigue during training of body joints (e.g., the wrist). In applications of this kind, mainly associated to rehabilitation and sport, data from pH sensing can be specifically related to muscle fatigue as due to the contractile activity of the muscles and the build-up of acid in muscle cells during exercise [41]. The availability of pH information can be used to design the optimal rehabilitation therapy for the patient, personalizing it as due to several factors as age, gender or training level. 

Another great opportunity raising from the availability of the wearable physiological flex sensor is represented by its potential use for personalized approaches for medicine, especially for aged patients needing remote assistance [42]. In this case the combination of sweat-pH and joint bending information can be conjunctly acquired with the aim to use the flex signal to remotely guide the patient to proper position the wearable physiological flex sensor and the sweat pH analysis to directly monitor the patient health of state with respect to different possible problems (e.g., cystic fibrosis, dehydration, diabetes and cancer), depending on the specific needs of patient. 

## 5. Conclusions

We developed a crosslinked NFs pH-Flex Sensors platform able to monitor the deformation of a human body joint as well as the pH of the skin. The common sensitive element of the sensors leads to a simplified process flow reduced at two steps: the electrospinning of the sensitive material on the PDMS slab support and the optimized thermal treatment offering a good conductivity, optimal mechanical coupling with PDMS substrate and good water resistance. The piezoresistive part of the platform showed very good sensing performance, with gauge factors amounting to 45.84 in traction and 208.55 in compression mode. pH sensor exhibited two linear behaviors and two zones could be determined: the one in which all is good and the one in which the alert signal is given and more analysis are required. Thanks to the design of the device, the two signals can be monitored contemporaneously, offering an innovative, simple and convenient bisensing platform for, as example, physical activity monitoring. 

## Figures and Tables

**Table 1:** Compression and traction maximum angles for human body joints.

Joint	Compression [°]	Traction [°]
Ankle	25 +/− 5	45 +/− 5
Knee	2.5 +/− 2.5	135 +/− 15
Hip	12.5 +/− 2.5	110 +/− 5
Elbow	157.5 +/− 7.5	5 +/− 25
Shoulder	40 +/− 5	85 +/− 5
Wrist	75 +/− 15	70 +/− 20
Finger	0 + 1	95 +/− 5

**Table 2:** Values of Rct, Cdl and R obtained by the fitting of EIS curves through equivalent circuit.

pH	R_ct_ [kΩ]	C_dl_ [μF]	R [Ω]
2.5	5.27 +/− 1.7	120.6 +/−8.7	293.5 +/− 3.8
3.2	7.88 +/− 2.0	96.99 +/− 7.7	284.4 +/− 3.1
5.8	14.80 +/− 2.9	51.71 +/− 6.9	288.8 +/− 3.7
7.13	8.64 +/− 1.9	86.69 +/− 7.42	288.2 +/− 2.4
7.3	7.27 +/− 1.8	98.58 +/− 7.7	291.3 +/− 2.5

## Data Availability

Not applicable.

## Supplementary Materials

The following are available online at https://www.mdpi.com/article/10.3390/s21124110/s1, Figure S1: Response of the flexible sensor to bending cycles.
