# Supplementary material for: Electrospun PEO/PEDOT:PSS Nanofibers for Wearable Physiological Flex Sensors
Source: Sensors (Basel). 2021 Jun 15;21(12):4110. doi: 10.3390/s21124110 (PMC8232244; doi:10.3390/s21124110)
Supplement: Supplementary file 1 [file sensors-21-04110-s001.zip › sensors-1243855-supplementary.pdf]

Supporting Informations

The figure S1 reports the characterization of the flex sensor for several bending cycles. The angle of deformation for these cycles was 45°.

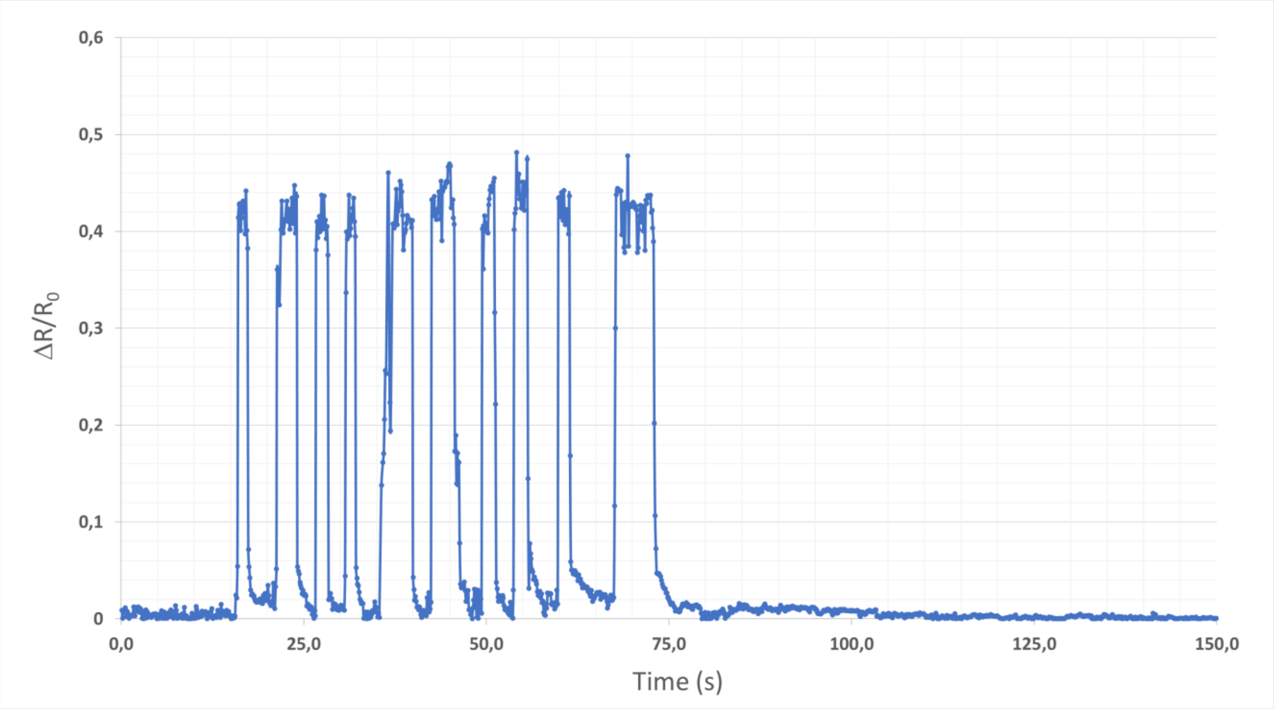

Figure S1. *Response of the flexible sensor to bending cycles.*
